# Supplementary material for: Unraveling neural complexity: Exploring brain entropy to yield mechanistic insight in neuromodulation therapies for tobacco use disorder
Source: Imaging Neurosci (Camb). 2024 Jan 9;2:imag-2-00061. doi: 10.1162/imag_a_00061 (PMC12224431; doi:10.1162/imag_a_00061)
Supplement: Supplementary Material [file imag_a_00061-supp.pdf]

Supplemental Materials

Urge to Smoke Results

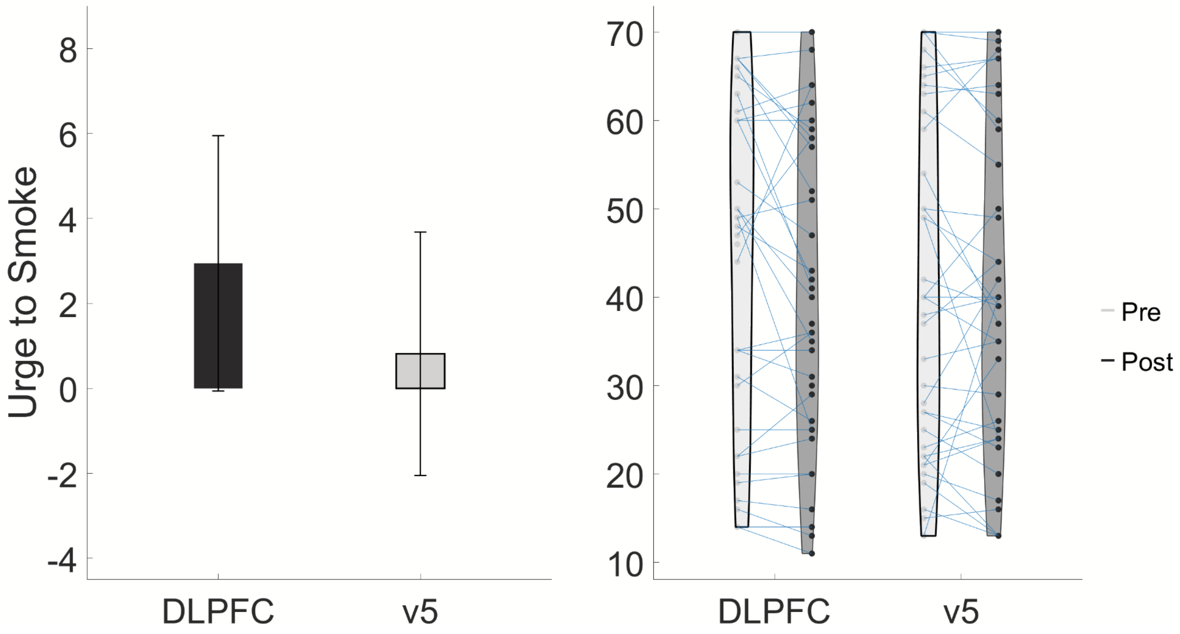

Figure S1 Changes in Urge to Smoke (related to Figure 3) No significant changes in Urge to Smoking were found for stimulation to left dlPFC.

Urge to Smoke Measurements			
		dlPFC	v5
UTS	Pre-rTMS	43.74 (20.05)	43.4 (19.54)
	Post-rTMS	41.1 (19.1)	41.7 (18.26)

Table S1 Urge to Smoke measurements (related to Table 1) Pre and Post-rTMS to both targets showing both their urge to smoke scores before and after treatment.

Sample Entropy of L/R Insula and left dlPFC nodes

Sample Entropy Pre-rTMS & Post-rTMS			
Schaefer Atlas Node	Time	dlPFC entropy, mean (SD)	V5 entropy, mean (SD)
35 (L Insula)	Pre-rTMS	1.516 (0.159)	1.528 (0.156)
	Post-rTMS	1.191 (0.193)	1.539 (0.17)
98 (L Insula)	Pre-rTMS	1.463 (0.114)	1.466 (0.123)
	Post-rTMS	1.324 (0.199)	1.442 (0.147)
99 (L Insula)	Pre-rTMS	1.464 (0.128)	1.481 (0.109)
	Post-rTMS	1.233 (0.162)	1.493 (0.143)
100 (L Insula)	Pre-rTMS	1.522 (0.119)	1.529 (0.111)
	Post-rTMS	1.246 (0.182)	1.567 (0.127)
143 (L Insula)	Pre-rTMS	1.533 (0.134)	1.553 (0.111)
	Post-rTMS	1.208 (0.141)	1.586 (0.139)
234 (R Insula)	Pre-rTMS	1.563 (0.135)	1.578 (0.12)
	Post-rTMS	1.26 (0.184)	1.586 (0.137)
235 (R Insula)	Pre-rTMS	1.515 (0.13)	1.528 (0.137)
	Post-rTMS	1.22 (0.186)	1.563 (0.151)
236 (R Insula)	Pre-rTMS	1.546 (0.14)	1.564 (0.14)
	Post-rTMS	1.245 (0.197)	1.596 (0.208)
302 (R Insula)	Pre-rTMS	1.596 (0.112)	1.59 (0.119)
	Post-rTMS	1.429 (0.182)	1.617 (0.131)

303 (R Insula)	Pre-rTMS	1.467 (0.113)	1.484 (0.112)
	Post-rTMS	1.347 (0.155)	1.484 (0.121)
304 (R Insula)	Pre-rTMS	1.537 (0.095)	1.544 (0.095)
	Post-rTMS	1.357 (0.158)	1.552 (0.13)
305 (R Insula)	Pre-rTMS	1.517 (0.129)	1.522 (0.129)
	Post-rTMS	1.308 (0.166)	1.567 (0.123)
340 (R Insula)	Pre-rTMS	1.527 (0.137)	1.525 (0.128)
	Post-rTMS	1.379 (0.144)	1.548 (0.141)
137 (L dlPFC)	Pre-rTMS	1.138 (0.129)	1.149 (0.114)
	Post-rTMS	1.083 (0.138)	1.13 (0.147)
138 (L dlPFC)	Pre-rTMS	1.167 (0.142)	1.17 (0.131)
	Post-rTMS	1.097 (0.147)	1.15 (0.134)
139 (L dlPFC)	Pre-rTMS	1.121 (0.105)	1.127 (0.1)
	Post-rTMS	1.088 (0.137)	1.119 (0.127)
140 (L dlPFC)	Pre-rTMS	1.142 (0.134)	1.158 (0.124)
	Post-rTMS	1.094 (0.134)	1.16 (0.119)
141 (L dlPFC)	Pre-rTMS	1.306 (0.146)	1.308 (0.14)
	Post-rTMS	1.152 (0.151)	1.348 (0.155)
142 (L dlPFC)	Pre-rTMS	1.15 (0.153)	1.153 (0.141)
	Post-rTMS	1.11 (0.135)	1.155 (0.124)

Table S2 Sample Entropy of each node Pre and Post-rTMS (related to Figure 5) Sample entropy measures for each node found to have significant changes in sample entropy post-rTMS to DLPFC. All measures are given as mean measures with standard deviation.

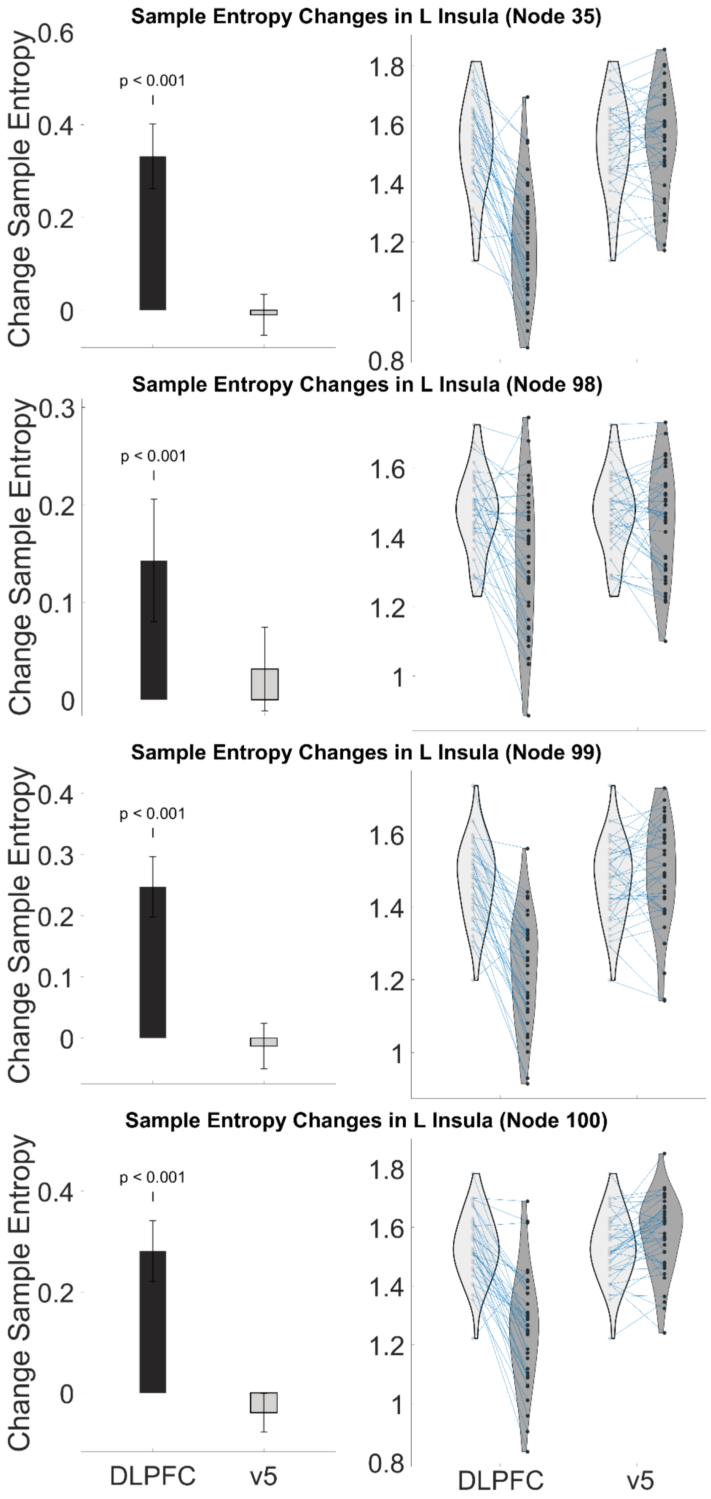

Figure S2 Stimulation to left dlPFC reduced sample entropy in left Insula (related to Figure 5) Plots show change group in sample entropy (left) and individual changes/distribution (right) for each left Insula node. Change values were calculated by subtracting Post-rTMS entropy values from Pre-rTMS values.

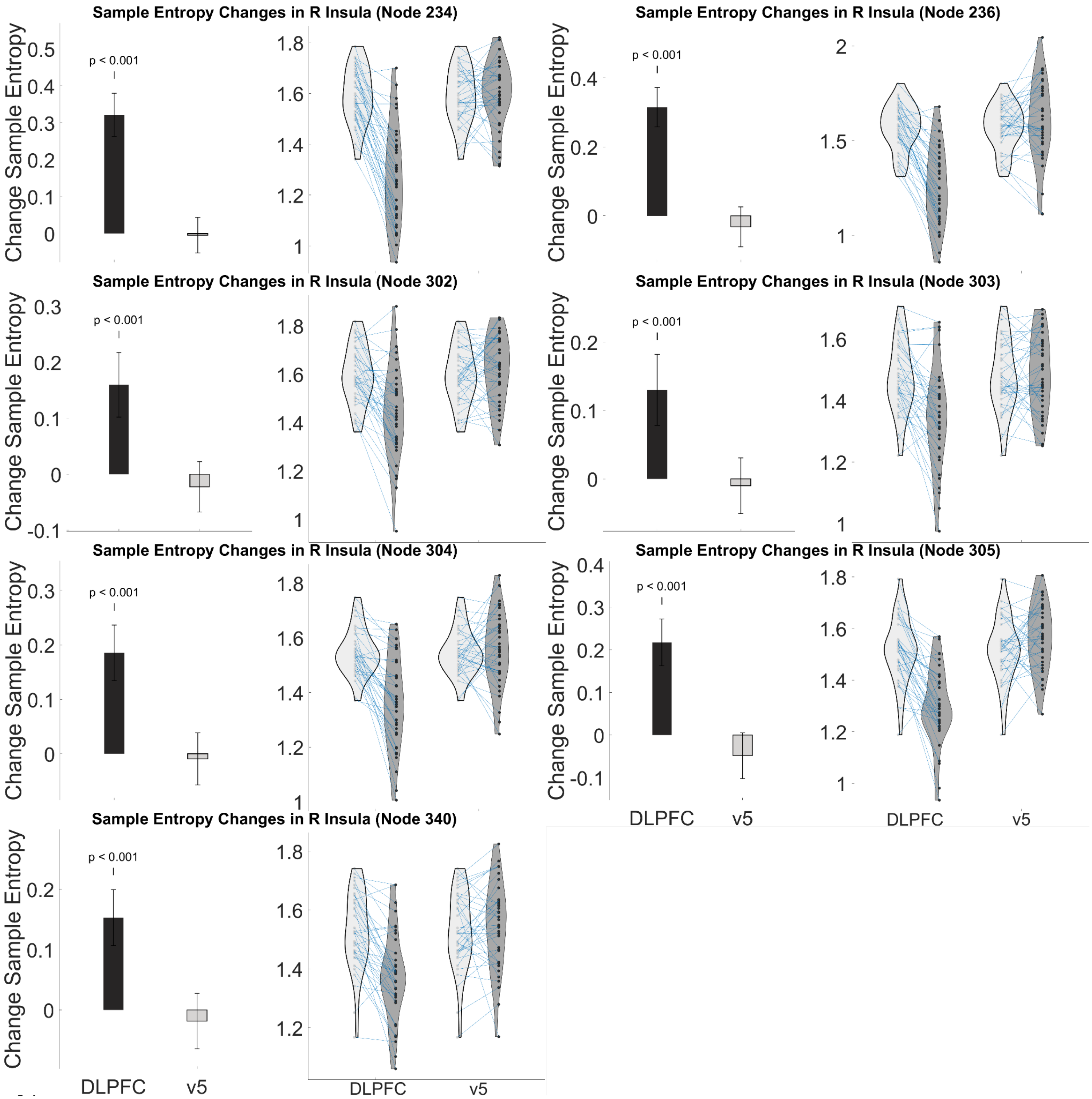

Figure S3 Stimulation to left dlPFC reduced sample entropy in right Insula (related to Figure 5) Plots show change group in sample entropy (left) and individual changes/distribution (right) for each right Insula node. Change values were calculated by subtracting Post-rTMS entropy values from Pre-rTMS values.

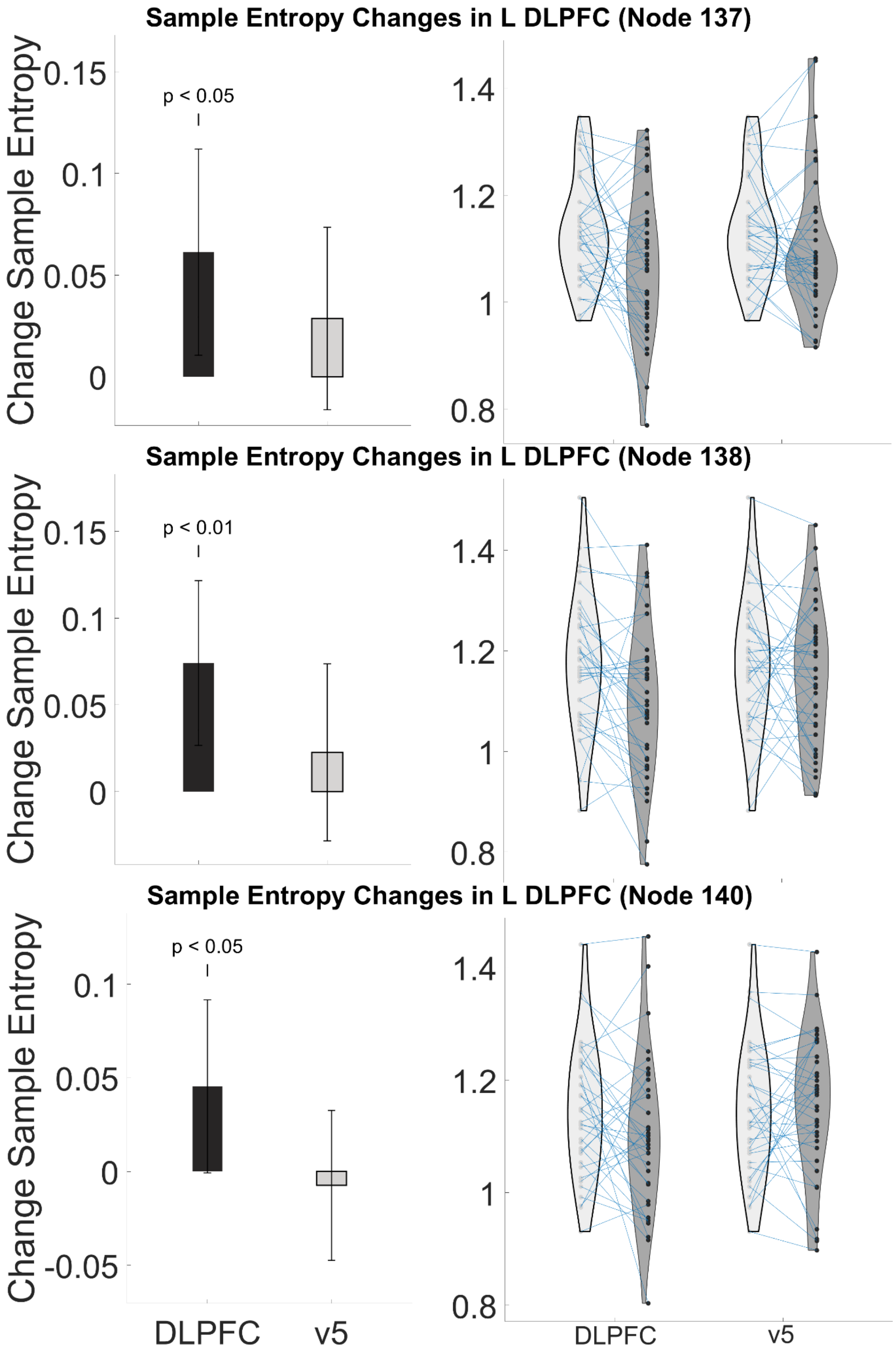

Figure S4 Stimulation to left dlPFC reduced sample entropy in left Insula (related to Figure 5) Plots show change group in sample entropy (left) and individual changes/distribution (right) for each left dlPFC node. Change values were calculated by subtracting Post-rTMS entropy values from Pre-rTMS values.

Confounding Variable Correlations

Sample Entropy across Ethnicity						
Node	Asian (N=5)	Native Hawaiian/ Pacific Islander (N=1)	Black / African American (N=11)	White (N=19)	Hispanic (N=4)	More than One Race (N=2)
35	1.6(0.2)	1.4(0)	1.5(0.2)	1.5(0.2)	1.5(0.1)	1.5(0.1)
98	1.5(0.1)	1.4(0)	1.5(0.1)	1.4(0.1)	1.5(0.1)	1.5(0.1)
99	1.5(0.1)	1.5(0)	1.5(0.1)	1.4(0.1)	1.6(0.1)	1.4(0)
100	1.6(0.1)	1.5(0)	1.5(0.1)	1.5(0.1)	1.5(0.1)	1.5(0)
143	1.6(0.1)	1.6(0)	1.5(0.2)	1.5(0.1)	1.5(0.1)	1.6(0.1)
234	1.6(0.2)	1.5(0)	1.5(0.2)	1.5(0.1)	1.6(0.1)	1.6(0)
235	1.5(0)	1.4(0)	1.5(0.2)	1.5(0.1)	1.5(0)	1.5(0)
236	1.6(0.1)	1.6(0)	1.5(0.1)	1.5(0.2)	1.6(0.1)	1.5(0.1)
302	1.7(0.1)	1.4(0)	1.6(0.1)	1.6(0.1)	1.6(0.1)	1.5(0)
303	1.5(0.1)	1.4(0)	1.5(0.1)	1.4(0.1)	1.5(0.1)	1.5(0.2)
304	1.6(0.1)	1.5(0)	1.5(0.1)	1.5(0.1)	1.6(0.1)	1.5(0)
305	1.6(0.1)	1.4(0)	1.5(0.2)	1.5(0.1)	1.5(0)	1.5(0)
340	1.6(0.1)	1.5(0)	1.5(0.1)	1.5(0.1)	1.6(0.1)	1.6(0.1)
137	1.2(0.2)	1.1(0)	1.1(0.2)	1.1(0.1)	1.1(0)	1.1(0.1)
138	1.3(0.1)	1.3(0)	1.1(0.2)	1.1(0.1)	1.2(0.1)	1.2(0)
139	1.1(0.1)	1.1(0)	1.1(0.1)	1.1(0.1)	1.1(0)	1.1(0)
140	1.2(0.1)	1.1(0)	1.2(0.2)	1.1(0.1)	1.2(0.1)	1.1(0.1)
141	1.4(0.1)	1.4(0)	1.3(0.2)	1.2(0.1)	1.4(0.1)	1.3(0.1)

142	1.3(0.1)	1.2(0)	1.1(0.2)	1.1(0.1)	1.3(0.1)	1.1(0.1)

Table S3 No significant differences in Pre-rTMS entropy between ethnicities

Ethnic groups were compared for Pre-rTMS sample entropy measures to determine if there were significant differences. No significant differences were found for any of the nodes. This table shows how many participants in each ethnic group were included in this study and their group's mean sample entropy with standard deviation for each node.

Node	Age		Years of Smoking		Education Level	
	r	p	r	p	r	p
35	0.03	0.83	0.03	0.84	0.00	0.98
98	0.18	0.26	0.18	0.27	-0.22	0.16
99	0.05	0.76	0.12	0.45	0.05	0.74
100	0.15	0.34	0.14	0.36	0.05	0.77
143	0.15	0.36	0.13	0.40	0.17	0.28
234	-0.01	0.95	-0.01	0.97	-0.07	0.66
235	0.03	0.87	0.02	0.88	-0.06	0.70
236	0.00	0.99	0.00	0.98	0.07	0.64
302	-0.07	0.67	-0.13	0.42	0.25	0.11
303	0.03	0.85	0.12	0.45	-0.19	0.22
304	0.18	0.26	0.24	0.13	0.07	0.65
305	-0.06	0.72	-0.05	0.77	-0.29	0.06
340	0.23	0.14	0.16	0.31	0.03	0.84
137	0.12	0.46	0.09	0.59	-0.16	0.32
138	0.08	0.60	0.08	0.62	-0.16	0.30
139	0.16	0.30	0.20	0.21	-0.25	0.10
140	-0.01	0.95	-0.03	0.87	-0.19	0.24
141	0.13	0.39	0.21	0.19	0.17	0.27

142	0.21	0.19	0.09	0.55	0.05	0.77

Table S4 No significant correlations between Pre-rTMS entropy and confounding variables Pearson correlations for three confounding variables (age, years of smoking, and education) were calculation for Pre-rTMS sample entropy measures to determine if there were significant correlations. No correlations were found for any of the nodes for any of the variables. This table shows the pearson correlation coefficient (r) and the p-value of each coefficient for each variable with pre-rTMS sample entropy measures.

Exploratory Findings Entropy Results

Sample Entropy Pre-rTMS & Post-rTMS			
Schaefer Atlas Node	Time	dlPFC entropy, mean (SD)	V5 entropy, mean (SD)
133 (L ITG)	Pre-rTMS	1.21 (0.14)	1.23 (0.13)
	Post-rTMS	1.14 (0.14)	1.22 (0.14)
314 (R SFG)	Pre-rTMS	1.36 (0.14)	1.37 (0.14)
	Post-rTMS	1.21 (0.17)	1.4 (0.16)
318 R SFG)	Pre-rTMS	1.32 (0.17)	1.32 (0.17)
	Post-rTMS	1.22 (0.18)	1.31 (0.17)

Table S5 Sample Entropy of each exploratory node Pre and Post-rTMS (related to Figure 7) Sample entropy measures for each node found to have significant changes in sample entropy post-rTMS to DLPFC. All measures are given as mean measures with standard deviation.

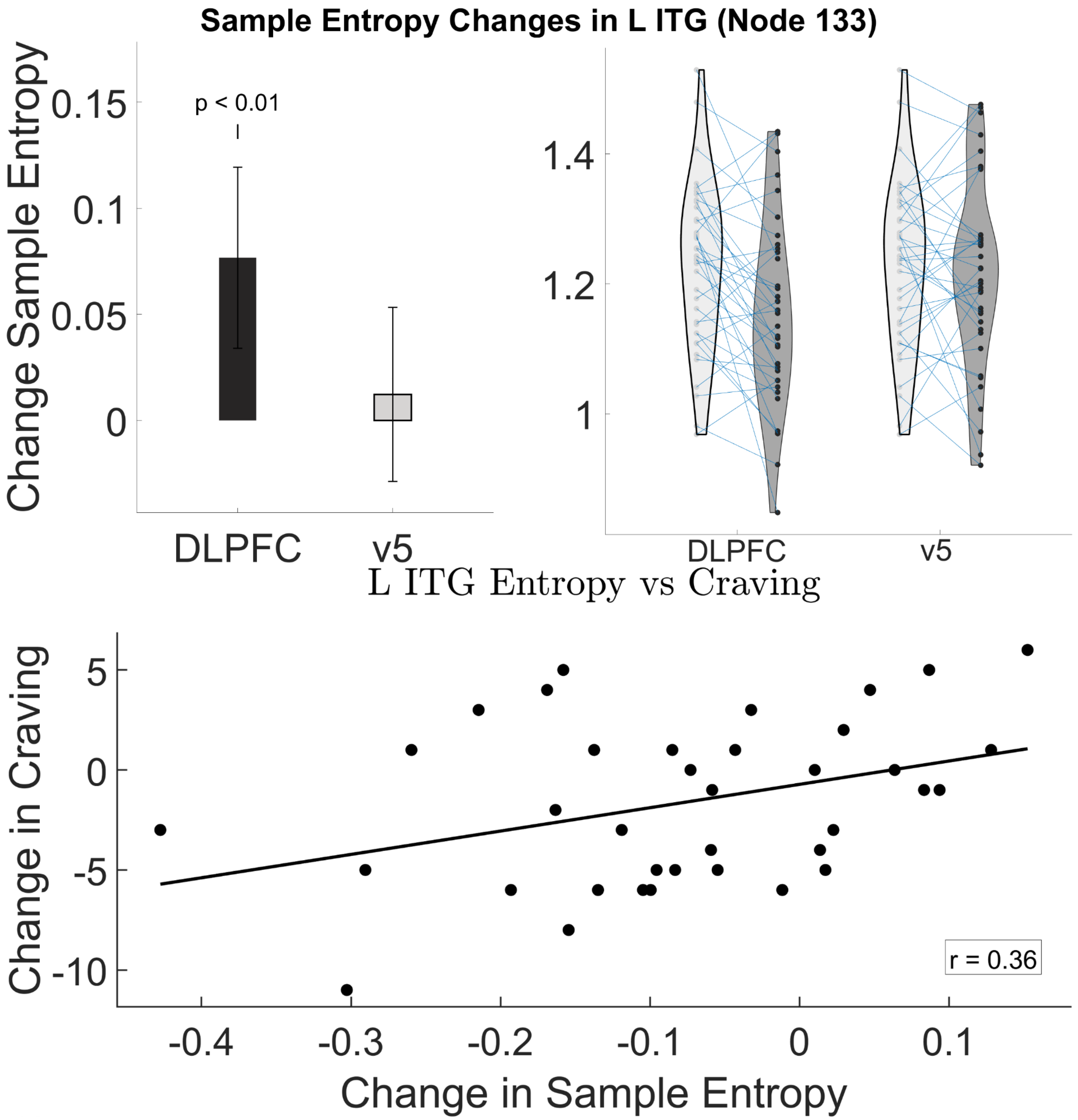

Figure S5 Stimulation to left dlPFC reduced sample entropy in left Inferior Temporal Gyrus (ITG) (related to Figure 7) Top: Plots show change group in sample entropy (left) and individual changes/distribution (right) for this node and the region that correlated with the node. Change values were calculated by subtracting Post-rTMS entropy values from Pre-rTMS values. **Bottom:** Correlation plot between changes in sample entropy in left ITG and craving (r=0.36, p= 0.027)as measured by the Shiffman-Jarvik Withdrawal Scale (SJWS) subscale for craving.

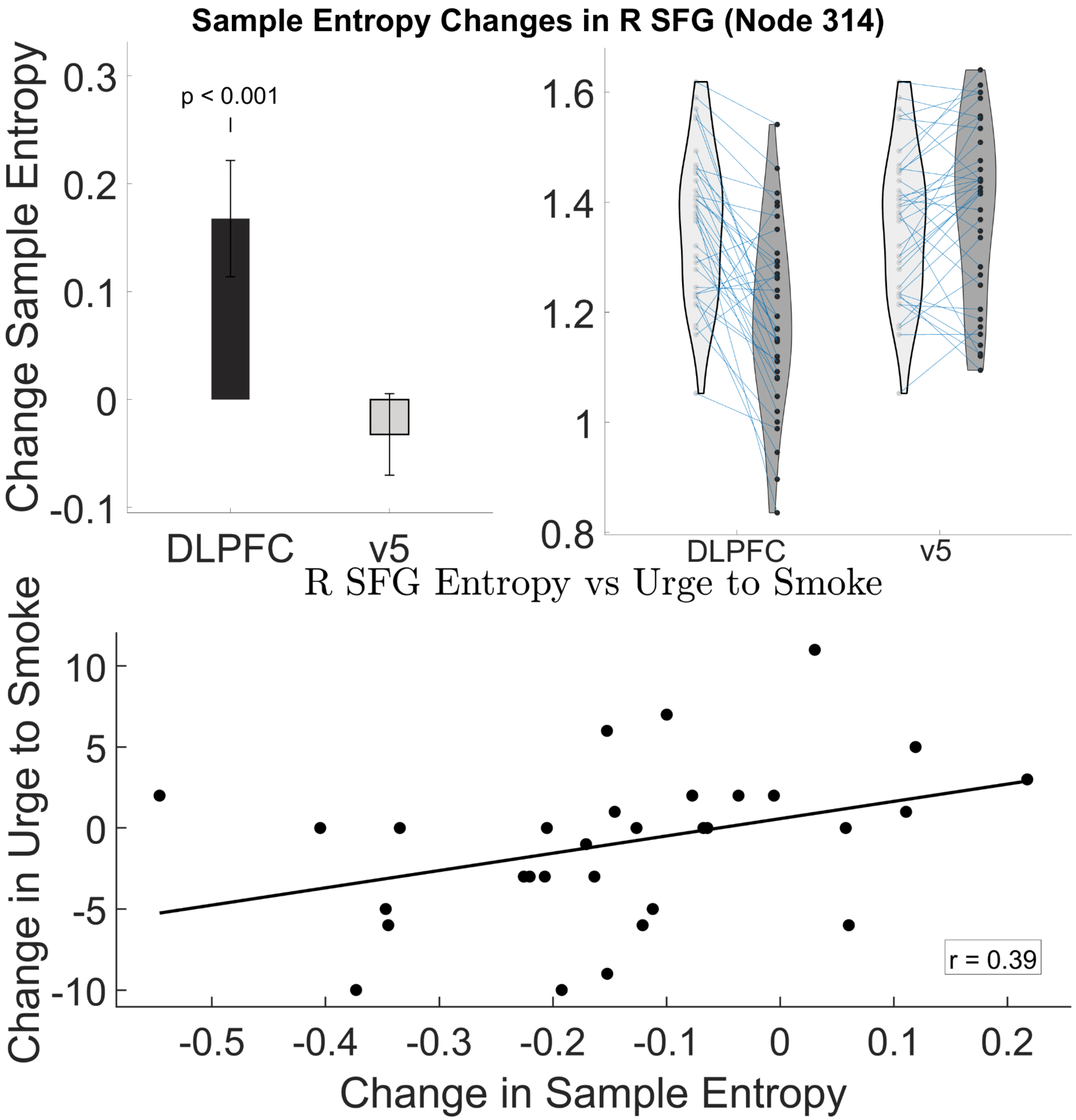

Figure S6 Stimulation to left dlPFC reduced sample entropy in right superior frontal gyrus (SFG)Top: Plots show change group in sample entropy (left) and individual changes/distribution (right) for this node and the region that correlated with the node. Change values were calculated by subtracting Post-rTMS entropy values from Pre-rTMS values. **Bottom:** Correlation plot between changes in sample entropy in right SFG and craving (r=0.39, p= 0.025)as measured by the Shiffman-Jarvik Withdrawal Scale (SJWS) subscale for craving.

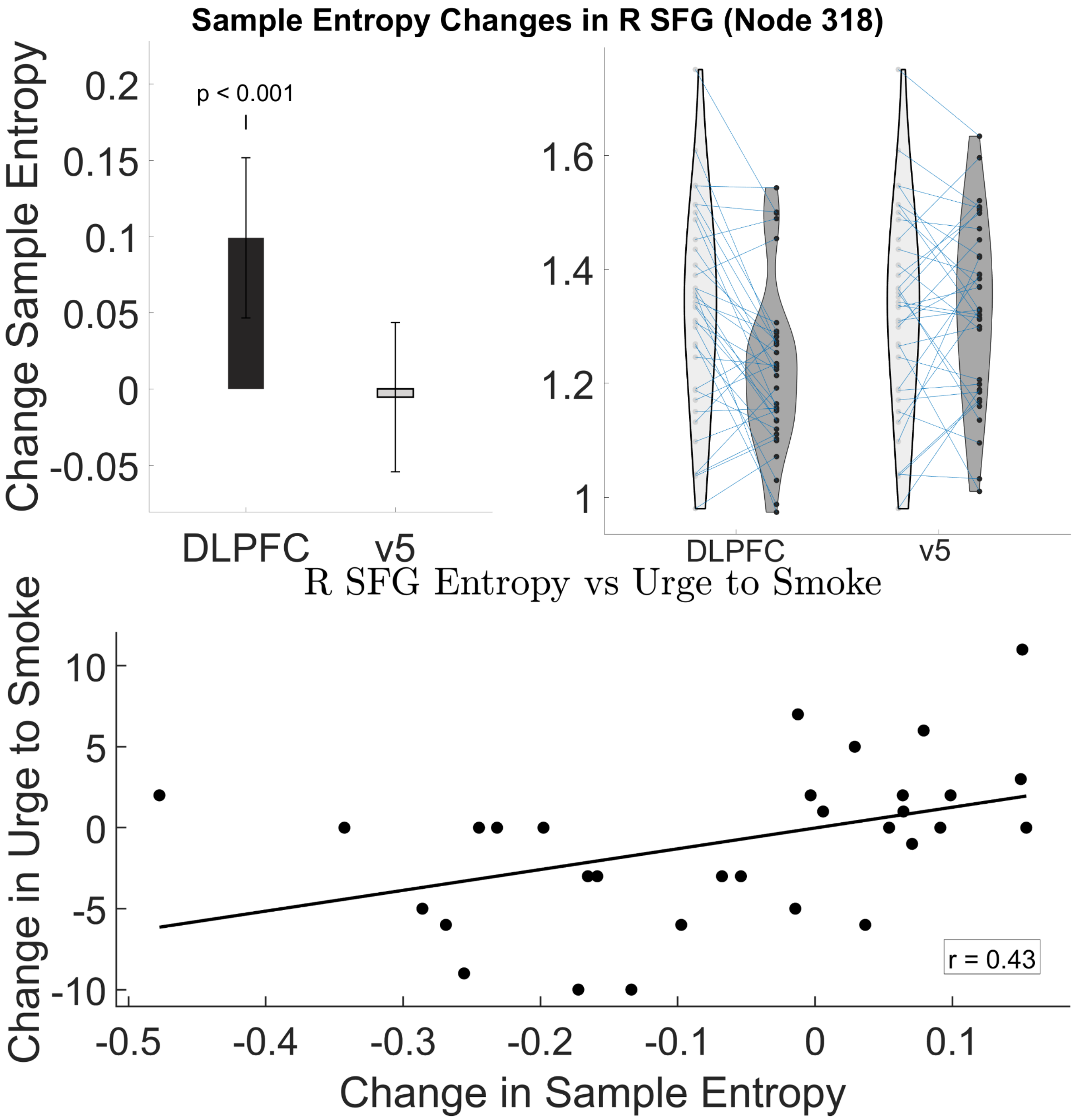

Figure S7 Stimulation to left dlPFC reduced sample entropy in right superior frontal gyrus (SFG) Top: Plots show change group in sample entropy (left) and individual changes/distribution (right) for this node and the region that correlated with the node. Change values were calculated by subtracting Post-rTMS entropy values from Pre-rTMS values. **Bottom:** Correlation plot between changes in sample entropy in right SFG and craving (r=0.43, p= 0.016)as measured by the Shiffman-Jarvik Withdrawal Scale (SJWS) subscale for craving.
